# Perineal Urethrostomy Enables Susceptibility of Bull Calves as a Natural Host Model for Bovine Trichomonosis

**DOI:** 10.3390/microorganisms13051070

**Published:** 2025-05-03

**Authors:** Katy A. Martin, Jenna E. Bayne, Krystal Chinchilla-Vargas, Sara L. Reece, Jeba R. J. Jesudoss Chelladurai, Tyler A. Harm, Jodi D. Smith, Douglas E. Jones, Courtney N. Blake, Matthew T. Brewer

**Affiliations:** 1 Department of Veterinary Pathology, Iowa State University College of Veterinary Medicine, Ames, IA 50011, USA; 2 Department of Clinical Sciences, Auburn University College of Veterinary Medicine, Auburn, AL 36849, USA; 3 Department of Pathobiology, Auburn University College of Veterinary Medicine, Auburn, AL 36849, USA; 4 Animal and Plant Health Inspection Service, United States Department of Agriculture, Ames, IA 50011, USA; 5 Practical Livestock Services, Casey, IA 50048, USA

**Keywords:** trichomoniasis, trichomonosis, sexually-transmitted disease, animal models of disease, cattle, parasitology

## Abstract

*Tritrichomonas foetus* is a sexually transmitted protozoan that causes early embryonic death in cattle. A challenge in trichomonosis research is that in vivo studies of treatments, diagnostic strategies, and vaccines are severely hampered by the logistical challenge and cost of maintaining adult bulls. Since natural infections are diagnosed in postpubescent animals, the paradigm is that only mature breeding bulls can be infected. In this study, we hypothesized that prepubescent bull calves could be artificially infected with *T. foetus* trophozoites for the purpose of conducting research trials. Initial attempts to directly infect bull calves with two different parasite isolates resulted in the sporadic and transient detection of parasite DNA but not culturable trophozoites. In vitro and in vivo studies suggested that urine directly inhibited trophozoites, likely by osmotic damage and mechanical flushing action. Studies utilizing a perineal urethrostomy to remove urine flow from the prepuce resulted in the ability to colonize the prepuce, with live organisms being cultured for as long as 15 days post-inoculation. Future studies optimizing this technique have the potential to accelerate the pace of bovine trichomonosis research and may have applications in the study of human trichomoniasis.

## 1. Introduction

*Tritrichomonas foetus* is a protozoan parasite of the bovine reproductive tract and the causative agent of bovine trichomonosis. Trophozoites are transmitted between bulls and cows during breeding, and there is no known environmental stage of the parasite. Bulls harbor the organism on the mucosal surfaces of the preputial cavity and generally remain asymptomatic [1]. Cows, on the other hand, typically develop clinical signs following infection. The motile trophozoites colonize the entire reproductive tract of the cow within two weeks [2]. Initial infection does not prevent conception in these animals, and few lesions are evident prior to 50 days of gestation [1,3]. After 50 days of gestation, mononuclear and neutrophilic inflammation occurs, leading to endometritis, cervicitis, and vaginitis of varying intensity depending on the immune response of the individual. The majority of infected cows experience fetal loss within the first five months of gestation [1,4]. The embryonic losses associated with *T. foetus* infection cause significant economic losses in the cattle industry [5,6].

Despite its importance, the life history of *T. foetus* in bulls remains poorly studied due to the cost and effort involved in maintaining large and potentially temperamental hosts. Due to the route of transmission, clinical infections are confined to breeding-age animals. The housing of mature cattle, particularly bulls in research settings, poses significant challenges in terms of facility availability, safety, and cost. Still, natural host models are preferred for disease studies as they provide more accurate information regarding immune response, pathology, and treatment. The lack of research progress regarding trichomonosis in bulls and the challenges associated with conducting studies with large and dangerous hosts have led to a significant knowledge gap. Our goal is to develop a more tractable animal model to study the biology of *T. foetus* and interventions for bovine trichomonosis. In working toward this goal, we hypothesized that prepubescent bull calves could be infected with the parasite.

There is no apparent structural parasite–host or receptor–ligand relationship that would preclude younger bulls from being infected with *T. foetus*. Competitive binding experiments have shown that *T. foetus* lipophosphoglycan (TF-LPG) inhibits the binding of *T. foetus* trophozoites to bovine vaginal epithelial cells (BVECs), indicating that TF-LPG mediates host–parasite interactions in cows [7,8,9,10]. For example, there was a 40–50% reduction in *T. foetus* binding to BVECs when 25–50 μg of *T. foetus* LPG was present [8]. However, the specific bovine host cell receptor for *T. foetus* binding has not been identified in either cows or bulls. The related human parasite, *Trichomonas vaginalis*, is known to bind galectin-1 on the host cell surface via terminal galactose residues on parasite LPG [11,12]. However, galectin-1 knockdown only mitigated binding by 20% [11]. Galectin-1 is encoded in the bovine genome, and puberty-driven gene expression is not known to occur [13]. If younger or prepubescent calves express galectin-1, it may be sufficient for *T. foetus* trophozoite adhesion. To our knowledge, this has not been investigated. Taken together, there is scant evidence for an age-related receptor–ligand relationship that *T. foetus* must overcome in order to infect younger animals.

Epidemiological studies indicate that older bulls (>3 years) are the primary source of infection for naive cows and heifers [14,15]. This evidence, in combination with histopathological observations, has led to the longstanding dogma that deep crypts in the prepuce of older bulls provide an ideal, low-oxygen environment for *T. foetus* trophozoites [15,16]. From a microbiology standpoint, this is a tractable explanation. Yet, the preputial environment of younger bulls is, arguably, also anaerobic. Thus, we hypothesized that the observation of older bulls with trichomonosis is largely a product of the simple fact that mature bulls are the most likely to engage in the only behavior that transmits the parasite: copulation. Under field conditions, this obviously limits the occurrence of bovine trichomoniasis to postpubertal animals. Altogether, there is not a known physiological, structural, or molecular reason that precludes bull calves from infection.

In this study, we hypothesized that we could avoid the logistical challenge of infecting mature breeding cattle by directly infecting prepubescent bull calves, thereby creating a natural host model for future research in the field. Specifically, we investigated the possibility that 6–8-week-old *Bos taurus* bull calves could be infected directly with *T. foetus* trophozoites.

## 2. Materials and Methods

### 2.1. Parasites

*Tritrichomonas foetus* trophozoites were maintained in trypticase–yeast extract–maltose (TYM) medium supplemented with adult bovine serum (10%, ABS) and 100× penicillin–streptomycin (1%, PS) (TYM-ABS-PS) [17]. Cultures were maintained at 35 °C and regularly sub-cultured to maintain cell concentrations of approximately 4 × 10^5^ trophozoites/mL. Cultures were maintained in sterile 15 mL centrifuge tubes filled completely with media and capped tightly to create an anaerobic environment. Experiment A calves received either lab (ATCC) or field (IA-1) strain parasites [18,19]. All other experiments in this study utilized IA-1 strain parasites. Studies were conducted under all applicable local laws and approved by the Iowa State University Biosafety and IACUC committees under protocols 18-217 and 21-264.

### 2.2. Animals and Experimental Infections

Cross-bred dairy bull calves (~70 kg) were purchased from a commercial dairy farm and infected at approximately 6–8 weeks of age. Twins or twin sibling calves were excluded from the study. Calves were group-housed indoors in a temperature-controlled environment. Four independent experiments were included in this study (experiments A–D). Experiment A consisted of 6 bull calves; experiments B, C, and D consisted of 3 bull calves each. The specific approach varied over the course of the study based on observations and outcomes of each experimental group. Specific methods for each experiment are presented separately below.

#### 2.2.1. Experiment A

**Figure 1:**
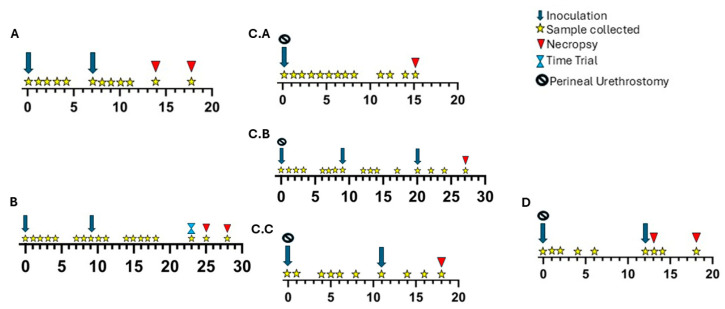
Overview of procedural timelines for experimental groups A–D. Experiment A included an initial inoculation on day 0 and a reinoculation on day 7. Experiment A calves were euthanized and necropsied on day 14 or day 18. Experiment B included an initial inoculation on day 0, reinoculation on day 9, and a third inoculation for a timed recovery trial on day 23. In experiment C, perineal urethrostomy surgeries and initial inoculations were performed on day 0. Calf C.A was euthanized and necropsied on day 15. Calf C.B was reinoculated on days 9 and 20 and euthanized and necropsied on day 27. Calf C.C was reinoculated on day 11 and euthanized and necropsied on day 18. In experiment D, perineal urethrostomy surgeries and initial inoculations occurred on day 0. Calves were reinoculated on day 12. Calf D.B was euthanized and necropsied on day 13 while calves D.A and D.C were euthanized and necropsied on day 18 post-surgery.

Experiment A evaluated the direct inoculation of the bull calf prepuce with two strains of *T. foetus* trophozoites. Six Holstein bull calves were inoculated with 1 × 10^8^ trophozoites suspended in 2 mL TYM culture medium on days 0 and 7. Parasites were delivered to the preputial cavity using a syringe and 12-gauge feeding needle. Three of the calves were inoculated with ATCC BP-4 Beltsville strain trophozoites (designated A.A, A.B, and A.C), and three were inoculated with IA-1 strain trophozoites (designated A.D, A.E, and A.F). Calves were sampled by instilling 0.5 mL of PBS into the preputial cavity via a pipette and syringe and applying negative pressure to recover fluid. Preputial samples were inoculated into InPouch TF^®^ (Biomed Diagnostics, White City, OR, USA). Samples were collected on days 0 (post inoculation), 1, 2, 3, 4, 7 (re-inoculation), 8, 9, 10, 11, 14, and 18. Calves were euthanized 14 or 18 days following the initial inoculation. Culture and fresh and fixed reproductive tract tissue samples were collected at the time of necropsy. An overview of the timeline of the study is shown in Figure 1A.

#### 2.2.2. Experiment B

In an effort to reduce the immediate leakage of inoculum from the prepuce, experiment B evaluated a collagen suspension matrix (PureCol^®^ EZ Gel, Advanced Biomatrix, Carlsbad, CA, USA) for inoculation. Three Holstein bull calves were inoculated with IA-1 strain trophozoites on days 0, 9, and 23. The initial inoculum was 4 × 10^8^ IA-1 strain trophozoites in TYM. Calves were re-inoculated with 2 × 10^8^ IA-1 strain trophozoites in a 50% collagen–TYM suspension. Lastly, a time trial was conducted on day 23, wherein calves were inoculated with 9.5 × 10^7^ IA-1 strain trophozoites in a 50% collagen–TYM suspension and preputial swabs were collected at 0 h, 0.5 h, 3 h, and 5 h post-inoculation. Preputial samples were collected using a TYM-soaked cotton-tipped applicator to swab the preputial cavity. Swabs were placed in 2.0 mL microcentrifuge tubes containing 1.5 mL TYM-ABS-PS supplemented with Modified Preston *Campylobacter* selective supplement (Oxoid LTD, Hampshire, UK). Calves were euthanized 25 or 28 days following the initial inoculation. Preputial swabs and fresh and fixed reproductive tract tissue samples were collected at necropsy. An overview of the timeline of the study is shown in Figure 1B.

#### 2.2.3. Experiment C

Based on the findings of experiment B, this experiment aimed to determine the in vivo impact of urine flushing on the establishment of infection with *T. foetus*. Three Angus–Holstein cross bull calves were included in this experiment (designated C.A, C.B, and C.C). Perineal urethrostomy procedures were performed to divert the urethra and urine flow from the preputial cavity to the perineum [20]. Under anesthesia (0.2 mg/kg xylazine IV), the perineal area over the ischial arch was clipped and aseptically prepared for surgery with chlorhexidine scrub and isopropyl alcohol. Lidocaine epidurals were performed to provide intraoperative analgesia. A 10 cm vertical incision on midline was made beginning approximately 5 cm ventral to the anus. The dense facial plane beneath the subcutaneous tissue was dissected using a combination of sharp and blunt dissection. The penis was identified via digital palpation, and the dorsal penile neurovascular bundle was bluntly separated from the underlying white dense fascia (tunica) surrounding the penis. The penis was transversely severed distally, and the stump sutured to the subcutaneous tissues and skin by horizontal mattresses, using 0 PDS, effectively diverting urine caudally, just below the ischial arch. The urethra was not spatulated due to diameter and visualization. The skin incision was closed in a simple continuous pattern using #1 nylon. All calves received meloxicam for postoperative pain control.

Following surgery, calves were immediately inoculated with 5.8 × 10^7^–6.3 × 10^8^ IA-1 strain trophozoites suspended in TYM media. Samples were collected using the same method as described for experiment B. Calves were euthanized on day 15, 19, or 27 (calves C.A, C.C, and C.B, respectively) following the initial inoculation. Cultures and fresh and fixed reproductive tract tissue samples were collected at necropsy. An overview of the timeline of the study is shown in Figure 1(C.A–C.C).

#### 2.2.4. Experiment D

The aim of experiment D was to confirm the results of experiment C and optimize the perineal urethrostomy procedure on bull calves. Three Angus–Holstein bull calves were included in this experiment (designated D.A, D.B, and D.C). Perineal urethrostomy procedures were performed as outlined for experiment C. Following the urethrostomy procedure, calves were immediately inoculated with 8 × 10^8^ IA-1 strain trophozoites in TYM, in a 2 mL total volume. Samples were collected using the same methods as described above for experiments B and C. Calves were re-inoculated on day 12, with 7.6 × 10^8^ IA-1 strain trophozoites. Calves were euthanized 13 (D.B) or 18 days (D.A, D.C) following the initial inoculation. Culture and fresh and fixed reproductive tract tissue samples were collected at necropsy. An overview of the timeline of the study is shown in Figure 1D.

### 2.3. PCR

Samples from experiment A were submitted to the Iowa State University Veterinary Diagnostic Laboratory for PCR testing. Following sample collection, InPouch TF^®^ pouches were incubated at 37 °C for 24 h and 200 μL of the media was removed and used to inoculate 1.5 mL of TYM media for culture purposes. The remaining InPouch TF^®^ sample was utilized for qPCR testing using the USDA-approved VetMAX^TM^-Gold Trich Detection Kit (Life Technologies, Austin, TX, USA) [21].

In experiments B–D, we opted to focus on using culture to demonstrate the recovery of live organisms rather than the amplification of DNA. In experiments B–D, we performed axenic culture in TYM media and conducted confirmatory PCR on culture-positive samples using previously published primers, TFR3/TFR4 [22]. DNA was extracted from 500 μL aliquots of positive cultures using the QIamp DNA Mini kit (Qiagen, Hilden, Germany), according to manufacturer instructions for cells grown in suspension. A negative culture sample was used as a negative control in these reactions. PCR was carried out in a 20 μL volume with 2 μL DNA template, 0.5 μM forward and reverse primers, and 1× Platinum^TM^ SuperFi^TM^ II PCR Mastermix (Invitrogen, Carlsbad, CA, USA). PCR conditions consisted of initial denaturation at 98 °C for 30s, followed by 30 cycles: 10s denaturation (98 °C), 10s annealing (60 °C), 1 min elongation (72°), and a 5 min step of final elongation (72 °C). Agarose gel electrophoresis was performed to confirm the amplification of bands of the expected size (350 BP).

### 2.4. Culture

**Figure 2:**
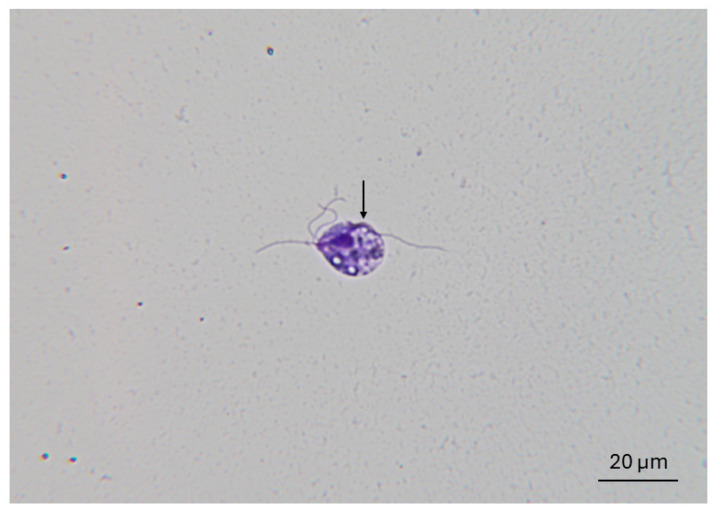
IA-1 strain *T. foetus* trophozoite recovered from TYM medium. Organisms were stained using Protocol^TM^ Hema 3^TM^ Fixative (Fisher Scientific, Hampton, NH, USA). Trophozoites were approximately 10–25 μm in length and are characterized by three anterior flagella and a prominent undulating membrane (arrow) which continues beyond the cell margin as a single posterior flagellum. (Magnification = 1000x).

Cultures were maintained at 35 °C and microscopically examined every 24 h for 7 days or until motile *T. foetus* trophozoites were observed. Cultures were considered negative if no live (motile) organisms were observed within 7 days. Organisms were identified morphologically; an example of a typical trophozoite observed in this study is shown in Figure 2. Trophozoites were approximately 10–25 μm by 5–15 μm. They possessed anterior flagella and a prominent undulating membrane continuous with the single posterior flagellum. Viable organisms demonstrated jerky, forward motion, typical of trichomonads. Positive cultures were verified by PCR.

### 2.5. Histopathology

Tissues of the reproductive system were collected from calves at the time of necropsy. Specifically, in experiment A, the prepuce, penis, seminal vesicles, bulbourethral gland, and prostate were collected. In experiments B–D, the prepuce and penis were collected. Tissue samples were fixed in 10% neutral buffered formalin (NBF). Following fixation, tissues were processed via routine histologic methods and embedded in paraffin blocks. Sections were cut from paraffin blocks, and H&E-stained with Signature Series^TM^ hematoxylin (Fisher Scientific, Hampton, NH, USA)) for five minutes and Richard-Allan Scientific^TM^ eosin (Fisher Scientific, Hampton, NH, USA) for one minute. Slides were reviewed by a board-certified veterinary pathologist (experiment A: JS; experiments C and D: TH). In successful experiments (C and D), the accessory sex glands were not collected since the perineal urethrostomy procedure eliminated the pathway for trophozoites to access these tissues. Experiment C and D tissues were scored for the following criteria of inflammation: epithelial degeneration, epithelial proliferation, intraluminal exudate, inflammation severity, inflammation distribution, hemorrhage, and edema. A total histologic score (THS) was calculated based on the sum of the individual criteria scores. Total histologic scores were categorized as no pathologic change (THS = 0), mild (THS = 1–5), moderate (THS = 6–10), marked (THS = 11–15), or severe (THS > 16).

### 2.6. Galectin-1 Immunohistochemistry

Immunohistochemistry was performed on tissues from experiment C and D calves to determine if galectin-1, a potential host cell receptor for *T. foetus*, was present. Bovine trachea and lung, known to express galectin-1, were used as positive control tissues [13]. Formalin-fixed, paraffin-embedded tissues (penis and prepuce) were cut and mounted on positively charged slides at a section thickness of 5 μm. Diva Decloaker (Biocare Medical, Pacheco, CA, USA, DV2004) in a decloaking chamber was used for antigen retrieval. Blocking was performed with Peroxidase Blocking Reagent (Biocare Medical, IPB5000G20) for 5 min and Background Punisher (Biocare Medical, IP974G20) for 5 min. Galectin-1 Recombinant Monoclonal antibody, JM13-37 (Invitrogen, MA5-32779), in DaVinci Green (Biocare Medical, PD900H) was used as the primary antibody at a 1:400 dilution with 2 h incubation. Rabbit-on-Canine HRP Polymer (Biocare Medical, RC542L) was used as the secondary antibody, with 30 min incubation. The signal was visualized using the DAB Chromagen Kit (Biocare Medical, IPK5010) following 5 min incubation. Tissues were counterstained with CAT Hematoxylin (Biocare Medical, CATHE-M) at a 1:10 dilution for 5 min, dehydrated in graded ethanol, and coverslipped.

### 2.7. Urine Co-Incubation Studies

Voided urine was collected, and an in vitro trophozoite viability assay was performed to determine the impact of urine presence on trophozoite survival. All urine samples used in the assays had a pH of 8.5 and specific gravity of ~1.03, which is within normal limits for bovine urine [23]. The assays were repeated three times in duplicate, using a urine sample from a different calf for each experiment. Trophozoites (1 × 10^4^) were incubated in a 1.5 mL total volume of TYM-ABS-PS supplemented with 0%, 5%, 10%, 25%, 40%, 50%, and 75% urine. Cultures were incubated at 35 °C under anaerobic conditions for 24 h. Trophozoites were enumerated via hemocytometer counts. One-way ANOVA with a Tukey post-test was used to determine statistical differences in trophozoite counts between urine concentrations.

## 3. Results

### 3.1. Sporadic and Transient PCR Positivity in Non-Urethrostomized Calves

In experiment A, calves were infected with either the IA-1 (“field strain”) or ATCC BP-4 Beltsville strain (“laboratory strain”) directly into the preputial cavity. Infection status was determined by conducting PCR on preputial samples incubated in InPouch TF^®^ commercial media. Standard qPCR diagnostic procedures detected parasite DNA transiently in all six calves on the day of infection, and in one calf 24 h post infection (Table 1). Interestingly, two calves were PCR-positive on day 7 (Table 1). However, none of these samples ever yielded trophozoites in culture. Thus, the same calves were re-inoculated, which resulted in qPCR positivity in four out of the six calves 24 h later, with sporadic PCR positivity 3–5 days post infection (Table 1). Again, we failed to culture trophozoites in TYM from any of these preputial samples.

### 3.2. Trophozoites Do Not Survive More Than 30 min in the Prepuce of Non-Urethrostomized Calves

**Figure 3:**
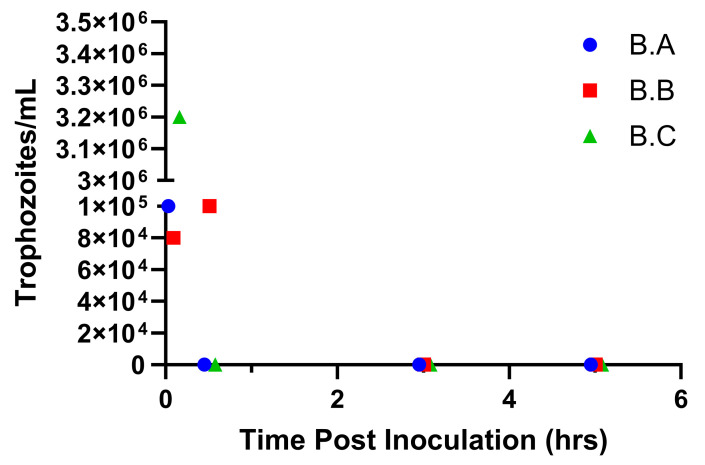
Viable trophozoites not recovered beyond 30 min post preputial inoculation in experiment B. After failure to isolate live trophozoites in experiment B, a timed recovery trial was conducted whereby calves were re-inoculated and then sampled at 0, 0.5, 3, and 5 h post inoculation. Motile trophozoites were recovered from 1 of 3 calves at 0.5 h post inoculation; live trophozoites were not recovered at any later timepoints. Data points indicate trophozoite counts from individual calves (B.A, B.B, and B.C), as represented in the figure legend.

A time trial was conducted (experiment B) by collecting serial samples from the prepuce of calves to temporally determine when parasites ceased to survive in the prepuce. Calves were sampled immediately following inoculation (0 h) and at 30 min, 3 h, and 5 h post-inoculation. Motile organisms were observed in cultures from all three calves immediately following inoculation and only in one of the three calves at 30 min post inoculation (Figure 3). No organisms could be cultured after 30 min of exposure to the preputial environment (Figure 3).

### 3.3. Trophozoites Are Killed Rapidly in the Presence of Urine

**Figure 4:**
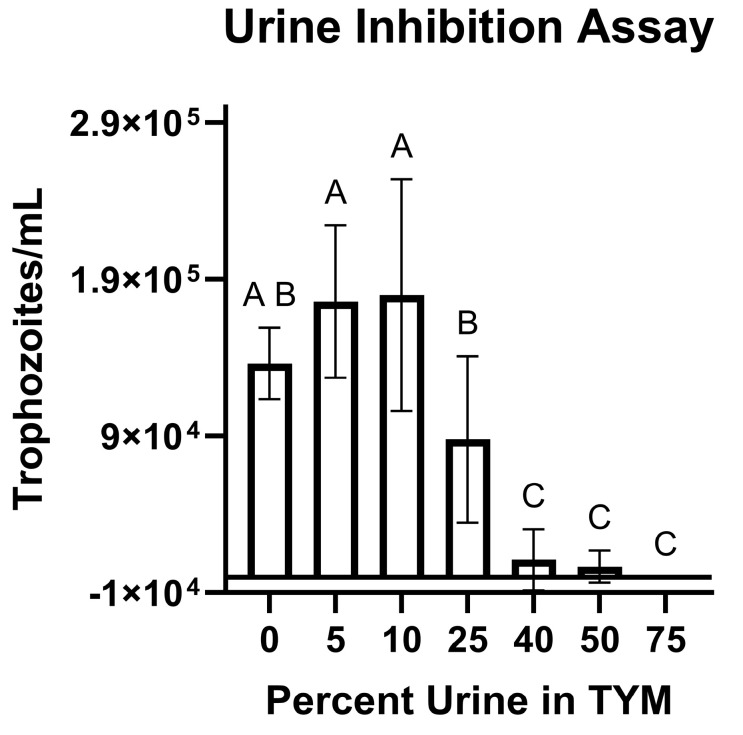
Urine inhibits *T. foetus* trophozoites in vitro. Trophozoites were cultured in TYM media supplemented with dilutions of calf urine. The presence of more than 25% urine resulted in inhibition of trophozoites in vitro. All counts were performed following 24 h at 35 °C under anaerobic conditions with a starting concentration of 10,000 trophozoites/1.5 mL. Bars indicate mean ± SEM of 3 independent experiments in duplicate using 3 separate bovine urine samples. Effect of urine concentration on trophozoite growth was determined using one-way ANOVA with Tukey’s post-test. Different letters (A, B, C) indicate statistical significance (α = 0.05).

We observed that DNA could be recovered for several days post-inoculation, while in contrast, we could only isolate live *T. foetus* parasites up to 30 min after inoculating calves (Figure 3). Thus, we hypothesized that urine was inhibiting parasite survival. In vitro viability assays using bovine urine samples demonstrated parasite death in the presence of more than 25% urine (Figure 4).

### 3.4. Putative Host Cell Receptor Galectin-1 Epitopes Are Present in the Reproductive Tract of Prepubescent Bull Calves

**Figure 5:**
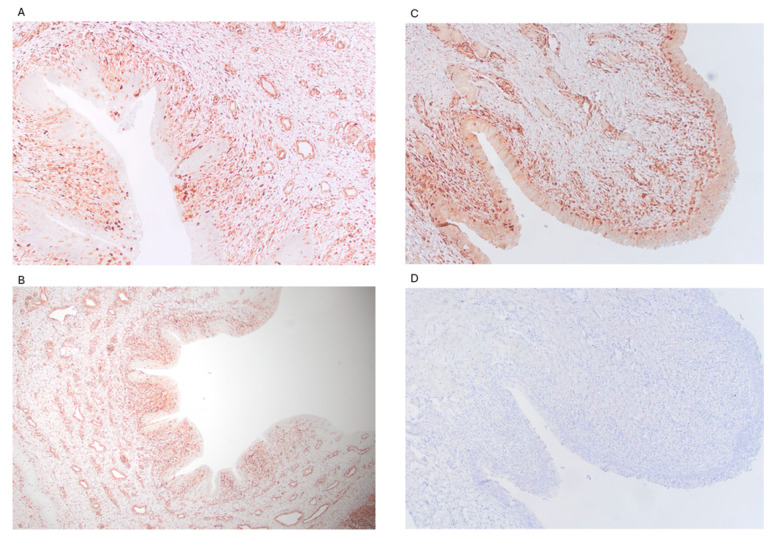
Galectin-1 is expressed by the penile and preputial tissues of pre-pubescent bull calves. All images were captured at 100x magnification. Immunohistochemistry using anti-galectin-1 antibody (JM13-37) demonstrates the presence of galectin-1 throughout the epithelium of sections of penis (**A**) and prepuce (**B**) of calves. Positive galectin-1 signal is indicated by peroxidase activity (red staining). Bovine trachea served as a positive control (**C**). Replacement of the primary antibody (JM13-37) with PBST served as a negative control (**D**).

Immunohistochemistry using a galectin-1-specific antibody confirmed the presence of galectin-1 protein in the penis and prepuce. This putative ligand for *T. foetus* adhesion was found throughout the epithelium and submucosa of both the penis and prepuce of prepubescent bull calves (Figure 5A,B). This is the first documentation of this galectin-1 epitope expression in reproductive tissues of prepubescent calves.

### 3.5. Perineal Urethrostomy Permits Survival and Growth of T. foetus in Bull Calves

Having observed the near-instantaneous inhibitory effect of urine on trophozoites, we hypothesized that the difficulty of infecting bull calves is due to the osmotic and flushing action of urine in the prepuce. To test this hypothesis, we used a standard urethrostomy procedure that re-routed the urethra to the perineum, resulting in a putatively urine-free prepuce.

**Figure 6:**
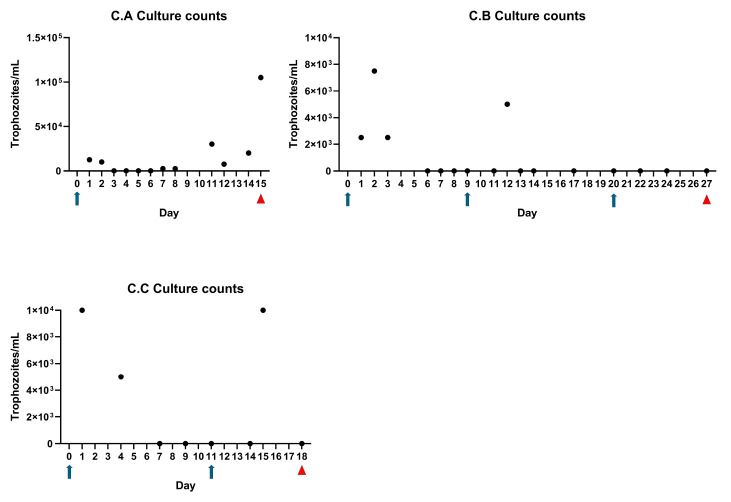
Recovery of viable trophozoites from urethrostomized calves in experiment C. Calves were urethrostomized and inoculated on day 0. All calves were culture positive for a minimum of 3 days following this procedure. Calf C.A was culture positive the day of necropsy (15 days post inoculation). Calves C.B and C.C were re-inoculated on days 9 and 11, respectively and calf C.B received a third inoculation on day 20. Individual dots indicate daily culture counts. Blue arrows indicate inoculation with parasites and red triangles indicate necropsy date. Calves D.A, D.B, and D.C. correspond to individual animals described in the materials and methods section.

**Figure 7:**
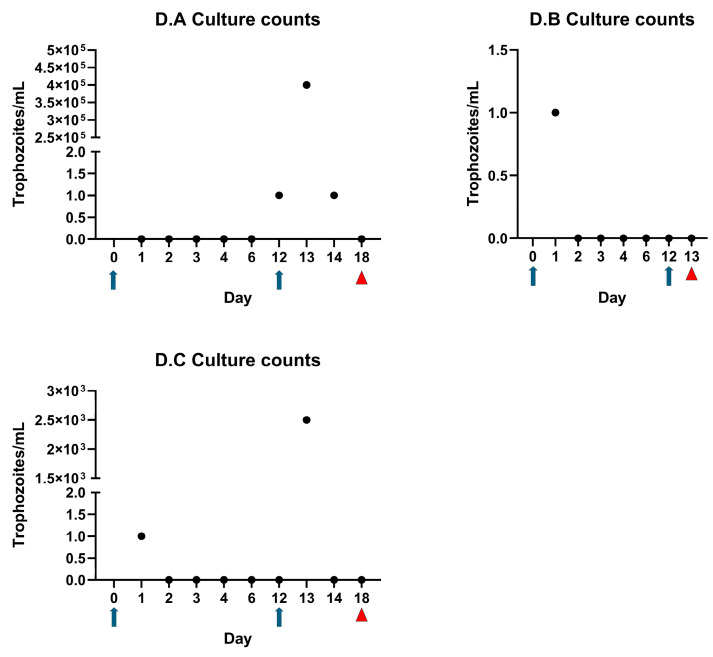
Recovery of viable trophozoites from urethrostomized calves in experiment D. Calves were urethrostomized and inoculated on day 0 (Blue arrows); 3/3 calves had live parasites for at least 24 h following perineal urethrostomy procedures and inoculation. Calves were re-inoculated on day 12; day 12 culture samples were collected prior to re-inoculation. Red arrows indicate necropsy date. Calves D.A, D.B, and D.C. correspond to individual animals described in the materials and methods section.

In both experiments C and D, we inoculated three calves following perineal urethrostomy procedures. Motile trophozoites were recovered from the prepuce of all six (100%) calves in these experiments 24 h or more following inoculation (Figure 6 and Figure 7). In experiment C, one calf (C.A) remained infected for 15 days, until the time of necropsy. The other calves in experiment C remained culture-positive for at least 3 days following inoculation (Figure 6). In experiment D, initial inoculation efforts revealed that live trophozoites were observed either 24 h (calves D.B and D.C) or 12 days later (calf D.A) (Figure 7). We attributed the relative success of experiment C to intraoperative macrolide antibiotics that dampened the systemic inflammatory response, whereas in experiment D, we wrongly assumed that we had perfected our technique and omitted intraoperative antibiotics. In experiment D, we performed a second inoculation on day 12 and recovered live trophozoites at levels that were orders of magnitude higher for at least 48 h after infection. This demonstrated again that the prepuce of urethrostomized calves is potentially a hospitable environment for *T. foetus* trophozoites (Figure 7).

Viable (motile, replicating) parasites recovered from cultures in experiments C and D were confirmed to be *T. foetus* via PCR. Thus, we did not recover another bystander flagellate. Altogether, the urethrostomy experiments demonstrated that the prepuce of prepubescent calves is suitable for *T. foetus* growth and survival following the removal of urine.

### 3.6. Inflammatory Cell Infiltrates Consistent with Trichomonosis Present in Infected Tissues

**Figure 8:**
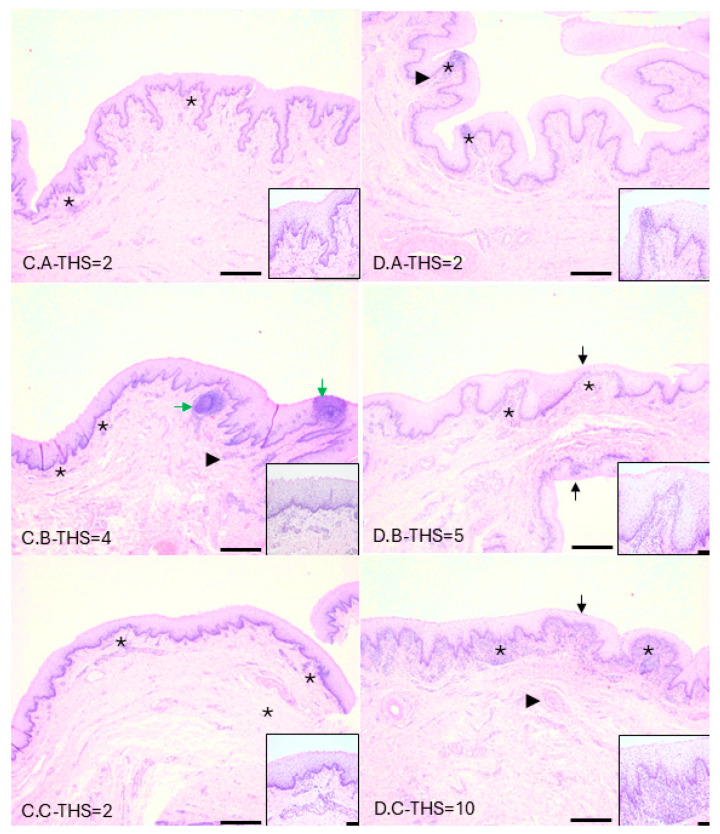
Mild to marked inflammation present in the preputial mucosa of experimentally infected bull calves. Mild histopathological changes were characterized by mild lymphoplasmacytic posthitis (asterisks, C.A, C.B, C.C, D.A, D.B), and prominent mucosal lymphoid nodules (green arrows). Moderate histopathologic changes consisted of mild to moderate epithelial attenuation (black arrows), lymphoplasmacytic to suppurative posthitis (asterisks, D.C), and mild scattered submucosal lymphoplasmacytic nodules and perivascular cuffs (arrowheads). Individual animal IDs are provided in the bottom left of each image as experimental group letter and calf designation letter (e.g., C.A). THS indicates the total histologic score. Scale bars = 500 μm (main images, 40× magnification) and 50 μm (insets, 200× magnification).

Tissues from experiment A were evaluated by an ACVP board-certified veterinary pathologist (JS). Histologically, these tissues were found to be within normal limits. Tissues from experiments C and D (penis and preputial mucosa) were evaluated and scored for inflammation by an ACVP board-certified veterinary pathologist (TH). Representative images are shown in Figure 8. Mild histopathologic changes (THS = 1–5) were observed in the preputial sections of five out of the six calves. These changes were characterized by mild lymphoplasmacytic posthitis, with one calf showing prominent mucosal lymphoid nodules and lymphoplasmacytic cuffing. Moderate histopathologic changes (THS = 6–10) were present in the preputial sections of one out of the six calves and were characterized by mild-to-moderate epithelial attenuation, lymphoplasmacytic to suppurative posthitis, and mild scattered submucosal lymphoplasmacytic nodules and perivascular cuffs. Mild histopathologic changes (THS = 1–5) were observed in the penile sections of one out of the six calves. These changes were characterized by mild lymphoplasmacytic urethritis and scattered lymphoplasmacytic nodules. Moderate histopathologic changes (THS = 6–10) were present in the penile sections of four out of the six calves. These moderate changes were characterized by scant intraluminal fibrinosuppurative exudate, mild epithelial attenuation, moderate lymphoplasmacytic-to-suppurative urethritis, and varying-sized lymphoplasmacytic nodules. Marked histopathologic changes (THS = 11–15) were present in the penile sections of one out of the six calves. Marked changes were characterized by moderate intraluminal fibrinosuppurative exudate, moderate epithelial attenuation, moderate-to-severe lymphoplasmacytic-to-suppurative urethritis, and varying-sized lymphoplasmacytic nodules. The inflammatory character of the urethritis was similar across all calves and was characterized by lymphocytes, plasma cells, macrophages, neutrophils, and scattered eosinophils. While sections of penis were reviewed, the importance of observed lesions and inflammation is less relevant as the penis was transected during PU procedures, rendering the tissue nonviable prior to parasite inoculation. Trophozoites were not observed in any histologic sections from this series of experiments.

## 4. Discussion

Uniformity is highly desirable when selling calves; it is well established that uniform groups of calves bring greater sale prices [24,25]. To take advantage of this, beef cattle operations and farmers strive to manage breeding periods that result in narrow calving windows and ultimately lead to groups of calves that are uniform in size. However, *T. foetus* causes early embryonic death and return to estrus which ultimately leads to a non-uniform calf crop [6]. A farm experiencing bovine trichomonosis for even a single breeding season can lead to the failure of a cattle enterprise. There are no approved treatments for *T. foetus* in the United States, and efforts to assess new drug treatments are severely hampered by cost and safety concerns associated with maintaining adult breeding-age bulls in a research setting [26,27]. This study was motivated by the need to advance the trichomonosis research agenda as we strive to study host–pathogen interactions, vaccines, diagnostics, and pharmaceuticals.

The conventional paradigm for *T. foetus* is that older bulls serve as carrier animals because trophozoites reside deep in the epithelial crypts of the prepuce, which becomes more prominent as bulls age [28]. In contrast, the anatomy of a juvenile bull’s prepuce is smoother, with less prominent crypts, and the penis is restricted to the preputial cavity via a frenulum that breaks down by 8–12 months of age [29]. It is assumed that the prepuce of older bulls is a more hospitable environment for trophozoites, wherein they reside in crypts that are optimally anaerobic [30]. Histological observation of *T. foetus* in the crypts led to the conclusion that they could not survive elsewhere in the male reproductive tract [31]. Epidemiological data demonstrating a higher prevalence of disease in mature bulls reinforce the notion that the deep crypts of older bulls are more hospitable for trophozoites, although it is logical that older bulls have bred more cows and are more likely to be infected. The hypothesis that *T. foetus* cannot survive outside the crypts has not been tested. Our study demonstrated that trophozoites could live within the prepuce of calves lacking deep epithelial crypts. However, we could not resolve the location of trophozoites histologically. This is likely due to the sloughing of parasites from the mucosal surface during the formalin fixation process in addition to few calves being culture-positive at the time of necropsy. Very few studies have ever shown *T. foetus* in the prepuce on routine histology, and others have reported failure to demonstrate organisms in culture-positive animals [31,32]. Without the visualization of trophozoites, it is possible although unlikely, that the inflammation and histologic changes observed in this study were related to the urethrostomy procedure rather than *T. foetus* infection.

During initial animal studies, we evaluated a simple direct preputial inoculation of non-urethrostomized, immunocompetent bull calves with trophozoites from both a laboratory and field strain of *T. foetus*. We did not attempt to immunosuppress calves during these studies. Our results demonstrated that we could recover parasite DNA via PCR but failed to demonstrate live parasites for several days following inoculation (Table 1). Next, we attempted to use PureCol^®^ EZ Gel (Advanced Biomatrix) polymerized collagen to reduce the loss of inoculum from the preputial cavity. This attempt was made due to our visual observation that liquid cultures leaked from the prepuce immediately following inoculation due to gravity. Based on temporal studies, we soon abandoned this tactic due to our observation that live parasites could not be recovered from the prepuce beyond 30 min following inoculation (Figure 3). This observation led us to hypothesize that urine was directly inhibitory for *T. foetus* trophozoites.

Urine inhibition assays were performed to determine the viability of trophozoites in the presence of urine. Our in vitro assays demonstrate a significant killing effect when the culture medium consists of 25% or more bovine urine. Taken with the observations that live parasites cannot be cultured from calves beyond 30 min of inoculation, and confirmation that galectin-1, a putative host cell receptor for *T. foetus*, is present in the reproductive tract of these calves, initial infection attempts (experiments A and B) likely failed due to the flushing action, osmotic pressure, or pH-related effects of urine. Additional studies are necessary to identify other host cell receptors for *T. foetus*.

Since parasites did not infect calves via simple direct inoculation, but were clearly inhibited by urine, we proceeded to attempt infections of bull calves following perineal urethrostomy. Perineal urethrostomy is a common procedure used to manage cases of urolithiasis in ruminants [20]. These infection studies demonstrated that the abrogation of urine flow allowed the colonization and growth of the parasite in the prepuce. Trophozoites could be cultured from all urethrostomized calves for at least 24 h and as long as 15 days following inoculation. Throughout this series of experiments, we emphasized the importance of culture-positive results over PCR-positive results. This is because a culture-positive result indicates the presence of viable organisms and therefore supports the establishment of a true infection, while a positive PCR result could be due to the presence of DNA of nonviable organisms. The survival of trophozoites, even for 24 h, is significant and demonstrates that the prepubescent bull calf model could be used for treatment studies.

Throughout these studies, we relied on culture results to determine infection status over PCR, as PCR cannot distinguish between the presence of viable and nonviable organisms. One drawback of culture as a diagnostic tool is the potential lag between sample collection and the identification of trophozoites in the sample. In many cases, trophozoites were not identified on the day of sample collection; rather, trophozoites were found following at least 24 h of incubation. Samples were evaluated for 7 days for the presence of organisms before being designated as negative. This lag time presented a challenge when determining whether or not to re-inoculate animals. Re-inoculations were performed in an effort to maximize the use of animals included in these studies and allow for additional opportunities to identify culture-positive samples. In experiment D, the day 12 sample collected prior to re-inoculation was found to be positive 3 days after sample collection. Ideally, re-inoculation would not take place until 7 days had passed and cultures had been confirmed negative; however, timing and cost constraints prompted us to reinfect some animals prior to this time point.

Challenging urethrostomized calves resulted in a vast and surprising improvement compared to initial attempts to infect the prepuce directly. This surgical procedure is typically well tolerated, even in feedlot settings, underscoring the feasibility of this technique for research purposes [20]. Alternative procedures, such as tube cystotomies, could also be investigated if a long-term (>1 month) infection model is necessary. Tube cystotomies have fewer complications in the long term and have the additional benefit of not interfering with penile anatomy [33,34]. The optimization of a specific perineal urethrostomy protocol is likely to enable more consistent outcomes. In an effort to reduce animal use, the minimum number of bull calves necessary to provide statistical power was utilized for these studies. Additional experiments are needed to optimize the model and improve consistency regarding infection duration in the bull calf model. In our work, related events (urine scald) of the perineum resulted in some inconsistencies in the necropsy schedule and data collected in experiments C and D. One difference between groups C and D, which may have impacted infection rates and durations, is the fact that animals in experiment C received preoperative antibiotics (tulathromycin), while animals in experiment D did not; it is possible that the antibiotic treatment of animals in experiment D may have improve infection rates or durations in these animals. While optimization will be the topic of future studies, the key finding of our present experiments is that *T. foetus* trophozoites can live in the prepuce of urethrostomized calves.

While it is true that organisms are more consistently recovered from older bulls, our study demonstrates that deep epithelial crypts are not a requirement for trophozoite survival, which has been held as a paradigm. In the context of our studies, it is important to note that calves and mature bulls differ with respect to the ultrastructure of the penis. Prior to puberty, the penis is restricted by a frenulum which fuses the prepuce and penis together, thereby preventing the exteriorization of the penis from the prepuce during urination [35]. As a result, flushing action by urine in the preputial cavity of calves occurs. Mature bulls, on the other hand, are not likely to flush the entire preputial cavity. The results of our galectin-1 immunohistochemistry and urine inhibition assay support the hypothesis that the toxic effect of urine in the prepuce prevents the establishment of infection with *T. foetus* in calves, rather than a lack of host cell receptor expression in prepubescent animals.

Beyond relevance in veterinary medicine, our results could have an application in human health. Human trichomoniasis, caused by the related pathogen *T. vaginalis*, is the most common non-viral sexually transmitted disease in humans [36]. *Tritrichomonas foetus* and *T. vaginalis* have similar clinical manifestations, with female hosts experiencing inflammation throughout the reproductive tract while male hosts act as asymptomatic carriers. Unfortunately, *T. vaginalis* is without a well-established animal model [37]. Interestingly, *T. foetus* infection in mice is frequently used as a model to test hypotheses about the human pathogen [38,39]. Besides mice not being a natural host, the mouse model has the challenge of being mainly used with female mice [38,40]. This is because administering intraurethral parasites is technically challenging, though it has been recently achieved [37]. Our findings demonstrate that a natural host model could be tractable for making predictions about human trichomoniasis caused by *T. vaginalis*. Future studies should explore the utility of the natural host calf as a model for *T. vaginalis* research.

The approach taken in this study had some limitations. For example, the cost of maintaining calves is more significant than that of maintaining mice. However, the present model represents a natural host model and is more cost-effective than maintaining adult breeding cattle. Still, the modest group sizes in this study did not allow us to pursue deeper questions about the model and will require additional investigation. There are several variables that should be explored further in the *T. foetus* bull infection model. One of these variables is cattle breed susceptibility, as this series of experiments utilized cross-bred animals. Infection studies using different strains and quantities of *T. foetus* trophozoites should also be performed to optimize this model. Our experiments primarily relied on a strain of *T. foetus* that was originally cultured from a bull in Iowa (IA-1). While early passage cultures were utilized for these studies, it is possible that the strain underwent some level of lab adaptation during the process of obtaining a pure culture from the original sample (preputial scraping). Evaluations of additional parasite strains would also be beneficial.

In summary, the series of experiments presented herein revealed that viable *T. foetus* trophozoites can be cultured from the prepuce of urethrostomized calves for up to 15 days. In contrast, viable organisms were not culturable from non-urethrostomized calves beyond 30 min post-inoculation. In vitro assays demonstrated the toxic effect of urine on trophozoites, leading to the hypothesis that the presence of urine in the prepuce of prepubescent calves prevented the establishment of infection. This study confirmed the presence of galectin-1, a potential host cell receptor for *T. foetus*, in the reproductive tract of bull calves. The ability to study *T. foetus* in a natural host without the safety risks and costs associated with housing mature bulls in a research setting is significant. This model will provide a safer and more economical method to study bovine trichomonosis in a research setting, allowing for advancements in the diagnosis, treatment, and prevention of disease. Lastly, a natural host model of a sexually transmitted trichomonad parasite has the potential to advance our understanding of the human parasite *T. vaginalis*, which currently lacks a suitable animal model. Significantly, our findings challenge the longstanding paradigm that deep preputial crypts are required for the infection of bulls.

## Figures and Tables

**Table 1:** Direct inoculation (Experiment A) of the prepuce of bull calves results in transient PCR positivity. qPCR using the VetMAX^TM^-Gold Trich Detection Kit (Life Technologies, Austin, TX, USA) was performed on *T. foetus* InPouch samples collected from bull calves following preputial inoculation. Positive indicates a CT value < 35, CT values ≥ 37 were considered negative and CT values of ≥35 <37 were designated as suspect. Positive, negative, and suspect CT values are shown in green, red, and yellow, respectively.

Day	A.A (ATCC)	A.B (ATCC)	A.C (ATCC)	A.D (IA-1)	A.E (IA-1)	A.F (IA-1)	Notes
	Negative	Negative	Negative	Negative	Negative	Negative	Pre-infection
0	Positive	Positive	Positive	Positive	Positive	Positive	Day of infection
1	Negative	Negative	Negative	Suspect	Positive	Negative	
2	Negative	Negative	Negative	Negative	Negative	Negative	
3	Negative	Negative	Negative	Negative	Negative	Negative	
4	Negative	Negative	Negative	Negative	Negative	Negative	
7	Positive	Negative	Negative	Positive	Negative	Negative	Prior to re-inoculation
8	Positive	Suspect	Positive	Positive	Suspect	Positive	
9	Suspect	Negative	Negative	Positive	Negative	Positive	
10	Positive	Negative	Negative	Suspect	Negative	Negative	
11	Negative	Negative	Negative	Negative	Negative	Negative	
14	Negative	Positive	Negative	Negative	Negative	Negative	Necropsy:A.A,A.D
18	NA	Negative	Negative	NA	Negative	Negative	Necropsy: A.B,A.C, A.E,A.F

## Data Availability

The original contributions presented in this study are included in the article. Further inquiries can be directed to the corresponding author.
